# Alkali-Activated Materials Doped with ZnO: Physicomechanical and Antibacterial Properties

**DOI:** 10.3390/ma16186224

**Published:** 2023-09-15

**Authors:** Agnieszka Ślosarczyk, Izabela Klapiszewska, Anna Parus, Olga Lubianiec, Łukasz Klapiszewski

**Affiliations:** 1 Institute of Building Engineering, Faculty of Civil and Transport Engineering, Poznan University of Technology, Piotrowo 3, PL-60965 Poznan, Poland; izabela.klapiszewska@put.poznan.pl (I.K.); lub.olga@wp.pl (O.L.); 2 Institute of Chemical Technology and Engineering, Faculty of Chemical Technology, Poznan University of Technology, Berdychowo 4, PL-60965 Poznan, Poland; anna.parus@put.poznan.pl

**Keywords:** alkali-activated materials, zinc oxide, slag, heat of hydration, antibacterial properties

## Abstract

The requirements related to reducing the carbon footprint of cement production have directed the attention of researchers to the use of waste materials such as blast-furnace slag or fly ashes, either as a partial replacement for cement clinker or in the form of new alternative binders. This paper presents alkali-activated materials (AAMs) based on blast-furnace slag partially replaced with fly ash, metakaolin, or zeolite, activated with water glass or water glass with a small amount of water, and doped with zinc oxide. The mortars were tested for flow, hydration heat, mechanical strength, microstructure, and antimicrobial activity. The obtained test results indicate the benefits of adding water, affecting the fluidity and generating a less porous microstructure; however, the tested hydration heat, strength, and antibacterial properties are related to more favorable properties in AAMs produced on water glass alone.

## 1. Introduction

Alkali-activated materials are an alternative to concrete made of Portland cement, and they have undergone dynamic developments in recent years. To obtain AAMs, an aluminosilicate precursor is needed; when activated with an alkali, it undergoes a polymerization reaction and gains mechanical properties in a much shorter time than a cementitious binder. The most commonly used precursors of aluminosilicates include metakaolin (MK), fly ashes (FAs), and ground-granulated blast-furnace slags (GGBFS) [1]. Aside from the content of elements such as Si or Al, aluminosilicate precursors also contain elements from the first group of the periodic table of elements (M). The ideal ratio of all components in the M:Al:Si ratio is 1:1:1.9. AAMs have significant potential due to their low energy demand, including production times and low temperatures, which relates to a low carbon footprint [2].

The mechanism of the reactions formed largely depends on the composition of the binder and the activator itself. Slag-based AAMs activated with sodium hydroxide have a three-step mechanism, while those activated with Na_2_SiO_3_ have a four-step mechanism. Among these four stages, the following can be distinguished: (i) dissolution, (ii) Si layer formation, (iii) CASH (calcium aluminate silicate hydrate) gel formation, and (iv) shell formation. Soluble in the structure, Si is introduced into the system through the water glass. In the initial stage, a high degree of saturation of such ions as Ca or Al and low Si can be observed. Ions present in large amounts quickly dissolve in the solution, releasing a large amount of heat. Si remains on the surface, the layer of which gradually builds up on the blast-furnace slag (BFS) surface in the second stage. The Si layer hinders the release of Ca and Al ions, slowing down the reaction and initiating the induction period, which proceeds to the third stage of the reaction. In the next, third stage, Ca and Al released earlier in the system react with Si to form a layer of CASH gel. As soon as the degree of undersaturation and the associated energy are higher than the activation energy, the Si layer dissolves, accelerating the formation of reaction products. In the last, fourth stage, the reaction products accumulate both on the surface of the BFS and in the solution. Creating shells on the BFS particles limits BFS dissolution [3].

Researchers are currently working on appropriate admixtures for AAMs to improve their physical and mechanical properties. The ongoing work on admixtures concerns water reducers and shrinkage-reducing admixtures [4]. Work is also underway to introduce oxides as an admixture to alkali-activated composites. These include TiO_2_ [5,6,7,8], SiO_2_ [9,10], Al_2_O_3_ [9], or ZnO [11]. The introduction of admixtures is associated with the acceleration of hydration and the increase in the products of this process, leading to a more compact structure and better mechanical properties (TiO_2_), reducing the permeability of samples (SiO_2_), or shortening the setting time (SiO_2_ and Al_2_O_3_) [9,12]. A few reports indicate the antibacterial use of AAMs doped with nanoparticles. Liu et al. [8] indicated that, in addition to photocatalytic properties, the admixture of TiO_2_ may also have antibacterial properties. A similar beneficial antimicrobial effect was indicated for the ZnO–SiO_2_ combination introduced into low-calcium AAMs based on fly ash. The beneficial antimicrobial effect is made possible by the free radicals produced, which eliminate unwanted microorganisms. An additional advantage of introducing nanoparticles into the composite matrix is their ability to propagate the hydration process via increasing the number of active centers affecting the growth of hydration products [8].

In search of antimicrobial properties, Kang and Ye [13] used alkali-activated slag modified with benzoate. Considering potential uses in wastewater infrastructure, the authors also examined the effect on the biological corrosion of this material. The alkali-activated slag slurries prepared by the authors, which were modified with sodium benzoate in the amounts of 1, 2, or 3 wt.% of GGBS, were characterized by a constant water-to-binder ratio of 0.4, and sodium hydroxide was introduced as an activator. The obtained test results indicate that an increase in the pH, a change in the concentration of sulfates, and the ratio of live/dead bacteria testify to the inhibition of the growth and progress of the colonization of sulfur-oxidizing bacteria. Compared to the paste sample without benzoate, a shallower depth and a denser layer of corrosion were observed, confirming the beneficial effect of benzoate. In their other work, Kang and Ye [14] modified alkali-activated slag with copper oxide and copper nitrate. They showed that introducing these materials increased the degree of hydration of the slag, and the material containing CuO showed 100% effectiveness in inhibiting the formation of biofilms and a shallower corrosion depth. The zinc used in this work [15] was an enrichment additive in alkali/zinc-activated fly ash nanocomposites produced using the hydrothermal method for dye removal and antibacterial applications. Fly ash activated with NaOH and zinc acetate formed a material with better adsorption of dyes and improved antibacterial properties.

There are few studies in the literature on the use of zinc oxide in AAMs. Taking the above factors into account, in this work, an attempt was made to introduce zinc oxide into a matrix containing combinations of binders of blast-furnace slag, fly ashes, zeolite, and metakaolin. Mortars were produced in two series: (i) containing only water glass and (ii) containing water glass and a small amount of water. The obtained mortars were characterized in terms of flowability, mechanical properties, microstructural properties, and hydration heat released during polymerization, comparing not only the effect of the components used but also the introduced water. The final aspect of this research comprised an assessment of the antimicrobial properties of the produced AAMs.

## 2. Materials and Methods

In the present study, blast-furnace slag (BFS), fly ash (FA), metakaolin (MK), and zeolite (Zeo) were used as low-emission alkali-activated binders. Quartz sand was used as aggregate, and zinc oxide (ZnO) was used as an admixture. The blast-furnace slag came from Lafarge Cement S.A. (Warszawa, Poland), the fly ashes were a by-product of hard coal combustion (Opole plant, Opole, Poland), and the MK and Zeo are MK-40 and Z-50, respectively, products of ASTRA (Straszyn, Gdańsk, Poland). The binders used were subjected to an assessment of the oxide composition (XRF analysis), presented in Table 1. Commercial zinc oxide, CAS number 1314-13-2 (Merck, Darmstadt, Germany), and a standard aggregate (quartz sand) with grain size < 2 mm from Kwarcmix (Tomaszów Mazowiecki, Poland) were used in the tests. The physical properties of all components used in this study are presented in Table 2.

The AAM composites were produced in two research groups, “A” and “B”, the detailed ingredients of which are presented in Table 3. The base recipe is a system made of 450 g of slag with 450 g of water glass (group A) or 400 g of water glass and 50 g of distilled water (system B). Moreover, 0.1 wt.% of the ZnO admixture was introduced into each of the systems. Then, 30 or 50 wt.% of the slag was replaced with fly ash, zeolite (30 wt.%), or metakaolin (50 wt.%). For the purposes of comparison, 0.1 wt.% of ZnO was added to these systems as well. The procedure for obtaining AAMs was taken from the PN-EN 196-1 standard, where the methodology for obtaining cement composites was presented; this was also presented in detail in our earlier work [16]. The introduction of ZnO in group A consisted of dry mixing with an alkali-activated binder; meanwhile, in group B, a suspension was created with ZnO and distilled water using a magnetic stirrer (mixing for about 5 min), and the mixture was then poured into the mixing bowl. The produced samples in the form of bars with dimensions of 40 mm × 40 mm × 160 mm, twice compacted with 60 strokes, were de-molded after 24 h and stored in water at an ambient temperature until the test.

In order to assess the effect of the type of binder on the heat of hydration of the designed low-emission AAM mixtures, a 72 h test of the heat of hydration was carried out using the semi-adiabatic method, following the PN-EN 196-9 standard, using a semi-adiabatic calorimeter (TESTING Bluhm and Feuerherdt GmbH, Berlin, Germany). The test consisted of recording temperature differences between the test sample and the reference sample based on the amount of released heat that was determined. Each of the produced mixtures was subjected to a flow test using a flow table following the PN-EN 1015-3 standard. This test measured two perpendicular diameters of the fresh spread produced after 15 shocks of the shaking table. In the next stage, the mechanical properties of the obtained composites after 7 and 28 days of curing were determined. Flexural and compressive strength were tested using a MATEST ServoPlus Evolution testing machine (Matest, Treviolo (BG), Italy). For each flexural strength test, six bars were used, from which 12 samples were obtained for testing the compressive strength. The microstructure was assessed on mature samples using a VEGA3 scanning electron microscope (SEM) (TESCAN, Brno, Czech Republic).

The final aspect of the research comprised an evaluation of antibacterial properties. Microbiological purity was evaluated using the contact plate and by measuring the change in the optical density of the medium using methods analogous to those described in [17].

The method used to measure the change in the optical density (OD600) consisted of placing an analyzed sample (approximately 500 mg) into a sterile conical flask and adding 50 mL of sterile liquid tryptic soy broth (TSB). The samples were incubated for 24 h at 30 °C with constant shaking at a speed of 110 rpm; after this, the optical density measurements were taken at 600 nm using a spectrophotometer SYNERGY HTX multi-mode reader (BioTek, Winooski, Vermont, USA). Next, the microbial cell counts were calculated using the online calculator “*E. coli* Cell Culture Concentration from OD600 Calculator” [18].

The next method employed, the growth method, involved placing cement samples measuring 5 mm × 5 mm × 2 mm in Petri dishes filled with sterile tryptic soy agar (TSA). The plates were closed and incubated at 30 °C for 24 h. After this time, the plate was analyzed for grown colonies of microorganisms.

All tests were performed in three series of three repetitions.

## 3. Results

### 3.1. Heat of Hydration

**Figure 1:**
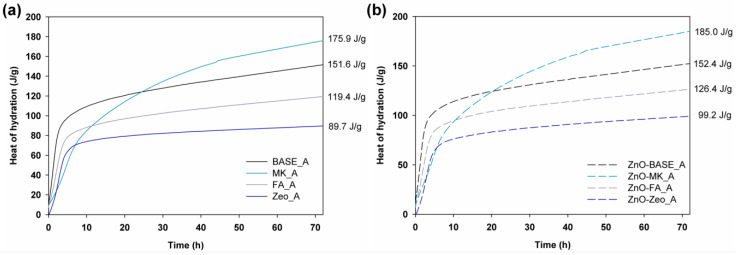
Heat of hydration curves of the AAM composites (**a**) doped with ZnO (**b**), prepared according to recipe “A”.

**Figure 2:**
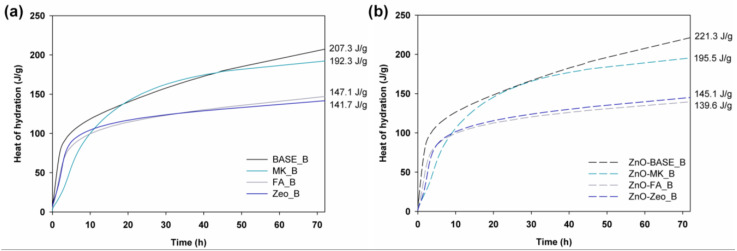
Heat of hydration curves of the AAM composites (**a**) doped with ZnO (**b**), prepared according to recipe “B”.

The semi-adiabatic heat of hydration study was carried out to determine the amount of heat released during the polymerization of the AAMs. The results of the 72 h test for series A composites are presented in Figure 1, and those for series B in Figure 2.

The presented curves showing the increase in the hydration heat over time for samples without the addition of ZnO (see Figure 1a) indicate that the most heat was released in the initial period from mixtures containing slag, slag and fly ash (30 wt.%), and slag and zeolite (30 wt.%). Replacing 50 wt.% of the slag with metakaolin leads to lower heat release in the initial period, which gradually increases over the entire range, reaching the highest value of 175.9 J/g after 72 h. For the remaining mixtures, the measured heat of hydration was 151.6 J/g (100% of slag), 119.4 J/g (30 wt.% of FA), and 89.7 J/g (30 wt.% of Zeo). When analyzing the effect of the admixture of 0.1 wt.% ZnO (see Figure 1b), it was observed that it either had no effect (the BASE sample) or caused an increase in the heat released of about 5% (ZnO-FA) or 10% (ZnO-Zeo and ZnO-MK). Interestingly, for mixtures made on water glass, the retarding effect of zinc oxide on the hydration process was leveled, which is indicated in the literature related to cement composites [17,19,20].

In the case of composites obtained according to the “B” recipe, higher values were recorded for the heat of hydration, both for the reference and ZnO-doped mixtures. The most significant increase related to introducing 50 g of water, replaced with water glass, was observed for the BASE mixture made of slag and MK, where the slag was partially replaced with metakaolinite. The introduction of water and ZnO did not affect the heat of hydration of the mortars based on fly ash and zeolite.

### 3.2. Flow Test

In order to assess the consistency of the alkali-activated mortars, a study of the spread on the flow table was carried out. The test results for both the A and B samples are summarized in Table 4.

Analyzing the obtained diameters for mixtures of the A series, it can be observed that the base sample containing 100% of the slag achieved a spread of 17.5 cm in the composites without the admixture of zinc oxide. The introduction of binder modifications resulted in a decrease (Zeo sample—16.5 cm) or a significant increase in the flow size obtained (MK sample—21.0 cm and FA sample—23.0 cm). The introduction of zinc oxide to the mortars resulted in an increase in the spreads obtained in the 3–34% range, depending on the binder’s configuration. Comparing the same configurations of binders in the samples obtained using method B (water glass + distilled water), a significant increase in the size of the spreads can be noticed, which, for the BASE sample alone, is 24.5 cm, an increase of 40%. The increases for the remaining MK, FA, and Zeo samples obtained using method B are 26, 20, and 18%, respectively. Additionally, in mixtures of the B series, an increase in the diameter of the obtained spread after adding ZnO can be observed. Depending on the configuration of the binders, changes are visible in the range of 4–23%.

### 3.3. Flexural and Compressive Strength

**Figure 3:**
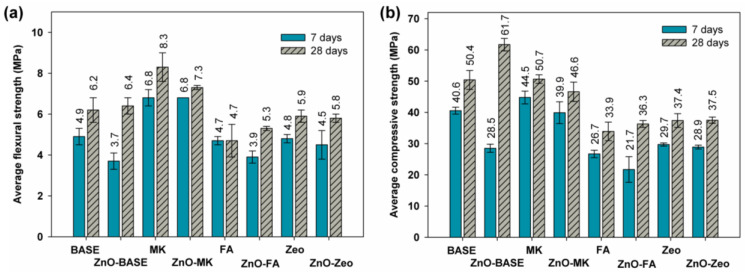
Average (**a**) flexural and (**b**) compressive strength of the “A” series AAMs.

**Figure 4:**
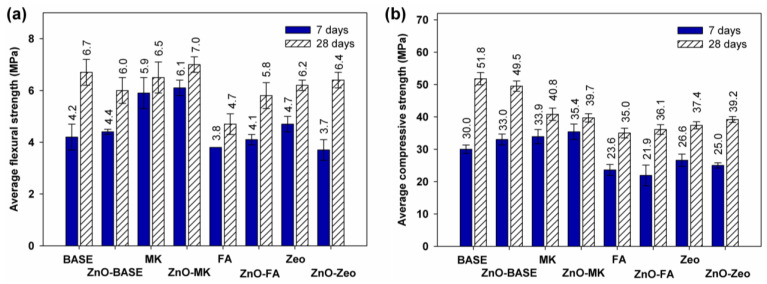
Average (**a**) flexural and (**b**) compressive strength of the “B” series AAMs.

The results of the mechanical strength tests after 7 and 28 days of curing are presented in Figure 3 for series A AAMs and Figure 4 for the mixtures of the B series.

The results of the average flexural strength of series A (see Figure 3a) indicate that the mixture with the highest strength after both 7 and 28 days is the MK sample, which reached values of 6.8 MPa after 7 and 8.3 MPa after 28 days of curing. The addition of ZnO in this binder configuration did not affect the initial 7-day strength, which, after 28 days, reached the value of 7.3 MPa. The composite with the lowest strength after 28 days was the FA-based composite, the average flexural strength of which was the same after 7 and 28 days. In the case of the average compressive strength (see Figure 3b), the composite with the highest average strength after 7 and 28 days among the recipes with different binder configurations was the MK mix (44.5 MPa and 50.7 MPa, respectively). Taking into account the influence on the strength of the admixture that was introduced to the composites after 28 days, the ZnO-BASE material reached the highest value of 61.7 MPa. As in the case of flexural strength, the lowest values of compressive strength were also achieved by the FA-based AAM.

The average flexural strength results for alkali-activated series B materials containing 400 g of water glass and 50 g of distilled water are shown in Figure 4a. After seven days of curing, the composite with the highest strength was MK, with a value of 5.9 MPa. The introduction of ZnO to the mortar did not change the flexural strength. After 28 days, the mix containing ZnO and metakaolin was characterized by the highest value of 7.0 MPa; slightly lower values were obtained for materials BASE (6.5 MPa), MK (6.5 MPa), and ZnO-Zeo (6.4 MPa). Analyzing Figure 4b, where the average compressive strengths for the series B composites are summarized, it was observed that the materials containing metakaolin are characterized by the highest strength values after seven days, both without (33.9 MPa) and with the addition of ZnO (35.4 MPa). However, these samples were not characterized by a sufficiently significant increase in strength over the next period of curing, so the materials with the highest strength after 28 days were the samples containing only slag, with values of 51.8 MPa (BASE) and 49.5 MPa for the ZnO-doped composite.

Comparing the series A and B samples, we observed that, in the case of flexural strength, the addition of distilled water had little effect on the increase in flexural strength of the BASE or Zeo and ZnO-FA composites. The compressive strength of the A series composites is higher than that of the B series for MK- and ZnO-doped systems: ZnO-BASE and ZnO-MK. The addition of water had no significant effect on the mechanical properties of AAMs with a combination of slag and zeolite (with and without ZnO) and slag and fly ash with an admixture of ZnO. The longer maturation time increased the strength in both the A and B series mixtures.

### 3.4. Microstructural Analysis

**Figure 5:**
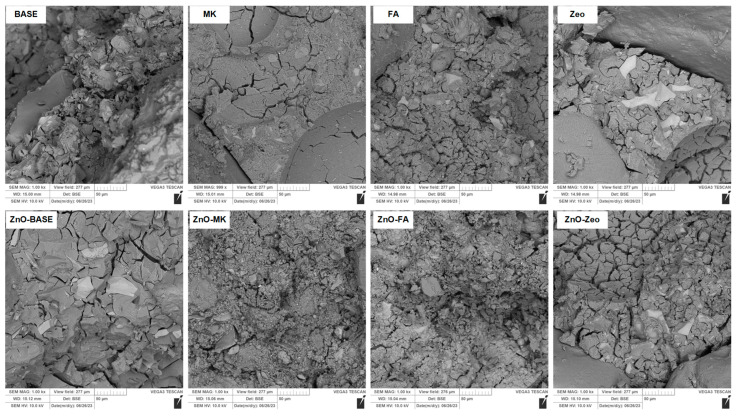
SEM images of “A” series AAMs.

**Figure 6:**
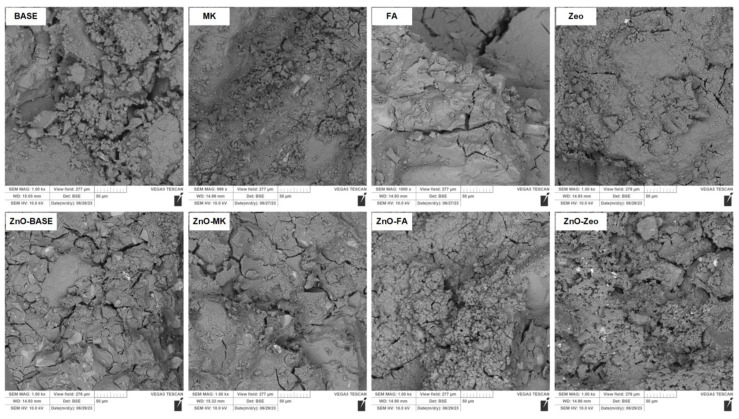
SEM images of “B” series AAMs.

The microstructure analysis was carried out based on the SEM images presented in Figure 5 (composites series A) and Figure 6 (composites series B).

The microstructure of undoped ZnO composites is compact, homogeneous, and devoid of clear air bubbles. Mortars made of water glass alone are more porous than the material obtained in the B series. In the case of samples containing fly ashes, single, unreacted ash grains are visible in both series.

In the case of materials doped with ZnO, the structure is densified. The presence of oxide particles increases the number of active centers, which favor the cross-linking of the structure. Comparing the SEM photos of composites without ZnO and those with an admixture, an additional layer surrounding the aggregate grains related to the “gelling” of the matrix can be seen.

### 3.5. Antimicrobial Properties

The antimicrobial properties were assessed using two methods; the results obtained for the optical density change are presented in Table 5 and Table 6 for the composites of series A and B, respectively. The results for microbiological purity, determined based on the growth of microorganisms on a glass plate, are presented in Table 7.

The most favorable systems regarding microbial purity were obtained by introducing zeolites, fly ashes, and metakaolin. The trend was observed in both methods—the smallest increase in microbial colonies (e.g., <8.0 × 10^6^ for FA_A and 6.51 × 10^7^ for Zeo_B) or their absence in the plate method. Unfortunately, doping with ZnO causes an increase in the tendency of microorganisms to grow. However, it should be noted that, for these samples (e.g., ZnO-MK_B—1.01 × 10^8^) compared to reference samples consisting of slag and water glass alone (BASE_B—3.97 × 10^8^), microorganisms developed nearly three times less than in the control sample. A significantly lower number of microorganism colonies was observed compared to the reference samples consisting of slag and water glass alone.

We observed the interesting phenomenon that the samples containing fly ash addition showed the highest microbiological purity. This is undoubtedly related to the composition of the fly ashes used to prepare the AAMs. Analyzing the amount of water glass introduced into the AAMs, it can clearly be seen that the composites into which a more significant amount of water glass was introduced (the samples in group “A”) showed lower microbiological purity.

## 4. Discussion

The presented research results concern AAMs without and with an admixture of ZnO produced entirely on water glass (the “A” series) or water glass with the addition of distilled water (the “B” series). Blast-furnace slag, fly ash, zeolite, and metakaolin in various proportions were used as binders. The use of this type of waste material is in line with the requirements of waste management, reducing CO_2_ emissions and thus reducing the carbon footprint of the materials produced [21].

Mohamed et al. compiled the values of the heat of hydration for various alkali-activated systems. They indicated that the water glass-activated material containing fly ash and granulated blast-furnace slag achieves heat of hydration values in the range of 118.0–140.0 J/g [22], which is close to the values presented in this work (119.4–151.6 J/g). When analyzing the effect of the partial replacement of slag with other binders, we found that only metakaolin caused an increase in the heat of hydration for systems containing water glass. A small addition of water reduces the heat of hydration of all the binder configurations. The introduction of the zinc oxide admixture is associated with a slight increase in the value of the heat of hydration, which is also visible in the case of cement composites [16].

The flow test values for the produced composites indicate that the addition of both water and zinc oxide increases the fluidity of the mixtures, which was also confirmed by the research of Zhang et al. [23], who studied the effect of various parameters on alkali-activated slag/fly ash composites. They observed that fluidity increased with increasing fly ash content in the mixture. In the case of cement mortars, similar observations were made by Kondraivendhan and Bhattacharjee, who obtained greater flow with increasing fly ash content for fly ash-blended cement mortar [24]. In the case of metakaolin in cement mortars, a decrease in the liquidity of the mixtures is noticeable, which may be related to the high water demand of the metakaolin itself [25]. It is also worth mentioning that the particle size of the binder matters; samples containing FA particles, i.e., a fine-grained binder, achieved the highest flow size.

The mechanical properties were more favorable for AAMs activated only with water glass. Considering the effect of metakaolin on the strength of the AAM composites, an improvement in the strength parameters was found, which is associated with a denser composite structure resulting from the presence of two interpenetrating networks of sodium and calcium aluminosilicates [25]. The introduction of nano-doping has a positive effect on strength. The admixture of nano-silica in low-calcium FA activated with water glass increased the strength by 19 and 75%, while it improved that of nano-alumina in AAM containing metakaolin by 33%. Introducing carbon nanotubes to low-calcium FA materials activated with water glass allowed us to achieve 70% higher strength, where metakaolin increased this parameter by 27%, and slag increased it by 19% [8]. The introduction of the metal oxide admixture is related to its influence on the material’s properties. It may also be related to certain limitations associated with it, such as a large specific surface area, which promotes the aggregation and agglomeration of particles, leading to difficulties with their even distribution and resulting in weak regions in the composite [8,9]. On the other hand, nanoparticles are places of growth and cross-linking of the composite structure, and depending on their type, they may favor or have a negative effect on the workability of mortars and pastes [8]. Undoubtedly, an additional aspect related to the introduction of nanoparticles may give the materials photocatalytic [26,27] and antimicrobial properties. Zinc oxide is known for its properties that inhibit the growth of microorganisms in cement composites [17,28,29,30]. In the case of AAMs, there are few reports in this area, which is why the presented research results are considered promising and worthy of further development. The introduced zinc oxide inhibits the growth of microorganisms in all tested areas compared to the sample made of blast-furnace slag alone. Adding a small amount of water has a beneficial effect on blocking the development of microorganisms on the surface of the composite.

## 5. Conclusions

The presented research results concern AAMs activated with water glass or water glass with a small addition of water based on blast-furnace slag. Materials with partial slag replacement by fly ash, metakaolin, and zeolite are also presented. Zinc oxide was introduced into all of the tested configurations, the purpose of which was to strengthen and impart antimicrobial properties to mortars. The analysis of the produced materials allowed the following conclusions to be drawn:

(i). AAMs made using the A method have a lower heat of hydration than those made using the B method. The introduction of zinc oxide leads to a slight increase in the heat of hydration of about 5% (ZnO-FA) or 10% (ZnO-Zeo and ZnO-MK), respectively;
(ii). The replacement of slag with zeolite reduces the diameters of the obtained spreads (from 17.5 cm to 16.5 cm), while metakaolin and fly ash contribute to increasing the diameter of the AAM spreads (up to 21.0 cm and 23.0 cm, respectively). The introduction of water and oxide admixtures to all of the analyzed configurations results in a larger spread diameter. The zinc oxide admixture can increase the flow size diameter of AAM mixtures of the A series in a range of 3–34% and 4–23% of the B series, respectively;
(iii). More favorable mechanical parameters were achieved via AAMs on water glass, and the highest strength (61.7 MPa) was achieved via the ZnO-BASE_A composite;
(iv). Materials with added water are characterized by a less porous structure; in all cases, the introduction of ZnO resulted in densification of the structure;
(v). The presented results for antibacterial properties are promising, and the modifications of the matrix with fly ash, metakaolin, and zeolite, as well as the introduction of the zinc oxide admixture, have a positive effect on inhibiting the growth of microorganisms.

The designed materials are a promising alternative to cement-based materials, enabling the use of low-emission binders in widely used civil engineering. The research presented in this paper confirms the proper selection of mixtures. In the future, the authors will focus on the durability of the designed mixtures, e.g., in freeze/thaw or carbonation tests and others.

## Figures and Tables

**Table 1:** XRF analysis results of basic precursors.

Sample	Oxide Composition (%)																		
	CaO	SiO_2_	Al_2_O_3_	MgO	SO_3_	K_2_O	TiO_2_	Fe_2_O_3_	MnO	SrO	V_2_O_5_	P_2_O_5_	ZrO_2_	Ag_2_O	Cr_2_O_3_	BaO	Eu_2_O_3_	SrO_2_	Rb_2_O
Slag	44.66	34.34	9.36	6.68	2.10	0.91	0.73	0.64	0.24	0.08	0.03	-	-	-	-	-	-	-	-
Metakaolin	0.61	52.19	42.59	-	0.17	0.39	1.58	1.95	-	-	0.04	0.35	0.06	0.06	-	-	-	-	-
Fly ash	5.34	50.46	29.21	1.57	0.94	2.32	1.53	7.08	0.06	0.14	0.05	0.98	0.05	0.09	0.03	0.11	0.06	0.14	-
Zeolite	6.76	70.03	11.50	0.93	-	6.14	0.41	3.42	0.05	0.07	-	0.44	0.04	0.12	-	0.07	-	-	0.03

**Table 2:** Physical properties of materials used in this study.

Parameter	Slag	Metakaolin	Fly ash	Zeolite	Quartz Sand	Admixture
Form	Powder	Powder	Powder	Powder	Grains	ZnO powder
Density (g/cm^3^)	2.91	2.69	2.10	2.27	1.58	5.68
Particle size distribution (nm)	79–164 1484–3580	190–342 3580–5560	122–712	91–190 2305–5560	0–2 mm	142–531
Polydispersity	0.908	0.633	0.899	0.963	1.000	0.052
Properties	Hydraulic	Pozzolanic	Pozzolanic	Pozzolanic	Aggregate	Antibacterial

**Table 3:** Sample composition.

Sample		Components (g)							
		Slag	FA	MK	Zeo	Water	Glass Water	Aggregate	ZnO
BASE	A	450	-	-	-	0	450	1350	0
ZnO-BASE		450	-	-	-				0.45
MK		225	-	225	-	0	450	1350	0
ZnO-MK		225	-	225	-				0.45
FA		315	135	-	-	0	450	1350	0
ZnO-FA		315	135	-	-				0.45
Zeo		315	-	-	135	0	450	1350	0
ZnO-Zeo		315	-	-	135				0.45
BASE	B	450	-	-	-	50	400	1350	0
ZnO-BASE		450	-	-	-				0.45
MK		225	-	225	-	50	400	1350	0
ZnO-MK		225	-	225	-				0.45
FA		315	135	-	-	50	400	1350	0
ZnO-FA		315	135	-	-				0.45
Zeo		315	-	-	135	50	400	1350	0
ZnO-Zeo		315	-	-	135				0.45

**Table 4:** Flow test results for all of the analyzed AAM composites.

Sample	A	B
	Flow Test (cm)	Flow Test (cm)
BASE	17.5	24.5
ZnO-BASE	23.5	25.5
MK	21.0	26.5
ZnO-MK	23.5	24.5
FA	23.0	27.5
ZnO-FA	25.5	28.0
Zeo	16.5	19.5
ZnO-Zeo	17.0	21.0

**Table 5:** Analysis of sample purity by measuring the optical density change (OD 600): “A” samples (the presented results are the average values of OD 600 and bacterial cells).

BASE_A	MK_A	FA_A	Zeo_A	ZnO-BASE_A	ZnO-MK_A	ZnO-FA_A	ZnO-Zeo_A
0.578	0.139	0.008	0.007	0.406	0.158	0.201	0.393
Number of bacterial cells in liquid samples [cel/mL]							
4.55 × 10^8^	1.10 × 10^8^	<8.0 × 10^6^	<8.0 × 10^6^	3.20 × 10^8^	1.09 × 10^8^	2.25 × 10^8^	2.99 × 10^8^

**Table 6:** Analysis of sample purity by measuring the optical density change (OD 600): “B” samples (the presented results are the average values of OD 600 and bacterial cells).

BASE_B	MK_B	FA_B	Zeo_B	ZnO-BASE_B	ZnO-MK_B	ZnO-FA_B	ZnO-Zeo_B
0.496	0.073	0.001	0.081	0.036	0.129	0.275	0.295
Number of bacterial cells in liquid samples [cel/mL]							
3.97 × 10^8^	5.93 × 10^7^	<8.0 × 10^6^	6.51 × 10^7^	2.88 × 10^7^	1.01 × 10^8^	1.69 × 10^8^	2.36 × 10^8^

**Table 7:** Purity analysis of AAM samples using the plate growth.

Samples	Microbial Growth	Samples	Microbial Growth
BASE_A	−/+	BASE_B	−/+
MK_A	−/+	MK_B	−/+
FA_A	−	FA_B	−
Zeo_A	−	Zeo_B	−
ZnO-BASE_A	−/+	ZnO-BASE_B	−/+
ZnO-MK_A	−/+	ZnO-MK_B	+
ZnO-FA_A	−	ZnO-FA_B	−
ZnO-Zeo_A	−	ZnO-Zeo_B	−/+

## Data Availability

Data will be made available upon request.
